# Novel perspectives on autophagy-oxidative stress-inflammation axis in the orchestration of adipogenesis

**DOI:** 10.3389/fendo.2024.1404697

**Published:** 2024-06-24

**Authors:** Chun Hong, Xinming Li, Kunli Zhang, Qiuyan Huang, Baohong Li, Haiyun Xin, Bin Hu, Fanming Meng, Xiangxing Zhu, Dongsheng Tang, Chuanhuo Hu, Chenyu Tao, Jianhao Li, Yang Cao, Hai Wang, Bo Deng, Sutian Wang

**Affiliations:** ^1^ State Key Laboratory of Swine and Poultry Breeding Industry, Guangdong Key Laboratory of Animal Breeding and Nutrition, Institute of Animal Science, Guangdong Academy of Agricultural Sciences, Guangzhou, China; ^2^ Institute of Animal Health, Guangdong Academy of Agricultural Sciences, Guangdong Provincial Key Laboratory of Livestock Disease Prevention Guangdong Province, Scientific Observation and Experiment Station of Veterinary Drugs and Diagnostic Techniques of Guangdong Province, Ministry of Agriculture and Rural Affairs, Guangzhou, China; ^3^ College of Animal Science and Technology, Guangxi University, Nanning, China; ^4^ Guangdong Provincial Key Laboratory of Animal Molecular Design and Precise Breeding, School of Life Sciences and Engineering, Foshan University, Foshan, China; ^5^ Guangxi Key Laboratory of Animal Reproduction, Breeding and Disease Control, Guangxi University, Nanning, China; ^6^ College of Animal Science and Technology, Hebei Agricultural University, Baoding, Hebei, China; ^7^ Branch of Animal Husbandry, Jilin Academy of Agricultural Science, Gongzhuling, China; ^8^ Chinese Academy of Sciences (CAS) Key Laboratory of Regenerative Biology, Guangzhou Institutes of Biomedicine and Health- Hong Kong University (GIBH-HKU) Guangdong-Hong Kong Stem Cell and Regenerative Medicine Research Centre, Guangzhou Institutes of Biomedicine and Health, Chinese Academy of Sciences, Guangzhou, China; ^9^ Division of Nephrology, Shanghai Ninth People’s Hospital, Shanghai Jiao Tong University School of Medicine, Shanghai, China; ^10^ Maoming Branch, Guangdong Laboratory for Lingnan Modern Agriculture, Maoming, China

**Keywords:** adipogenesis, autophagy, inflammation, oxidative stress, immune responses

## Abstract

Adipose tissue, an indispensable organ, fulfils the pivotal role of energy storage and metabolism and is instrumental in maintaining the dynamic equilibrium of energy and health of the organism. Adipocyte hypertrophy and adipocyte hyperplasia (adipogenesis) are the two primary mechanisms of fat deposition. Mature adipocytes are obtained by differentiating mesenchymal stem cells into preadipocytes and redifferentiation. However, the mechanisms orchestrating adipogenesis remain unclear. Autophagy, an alternative cell death pathway that sustains intracellular energy homeostasis through the degradation of cellular components, is implicated in regulating adipogenesis. Furthermore, adipose tissue functions as an endocrine organ, producing various cytokines, and certain inflammatory factors, in turn, modulate autophagy and adipogenesis. Additionally, autophagy influences intracellular redox homeostasis by regulating reactive oxygen species, which play pivotal roles in adipogenesis. There is a growing interest in exploring the involvement of autophagy, inflammation, and oxidative stress in adipogenesis. The present manuscript reviews the impact of autophagy, oxidative stress, and inflammation on the regulation of adipogenesis and, for the first time, discusses their interactions during adipogenesis. An integrated analysis of the role of autophagy, inflammation and oxidative stress will contribute to elucidating the mechanisms of adipogenesis and expediting the exploration of molecular targets for treating obesity-related metabolic disorders.

## 1 Introduction

It is widely acknowledged that adipose tissue performs a dual function, serving as a substantial energy storage organ, regulating the accumulation and release of energy, and as a pivotal endocrine organ contributing to systemic metabolism and preserving the organism’s homeostasis (1). Adipose tissue in mammals can be classified into two principal types: white adipose tissue (WAT) and brown adipose tissue (BAT). Moreover, white adipocytes can transdifferentiate into beige adipocytes, which exhibit similar morphological and functional characteristics to brown adipocytes, in response to stimuli such as exercise, cold exposure and other factors. The function of WAT is to store and release energy. Unlike WAT, BAT predominantly engages in thermogenesis, achieving efficient energy consumption via mitochondrial uncoupling. Beige adipose tissue exhibits a hybrid phenotype intermediate between WAT and BAT and can be stimulated to burn energy in response to β-adrenergic activation. Adipocyte hypertrophy (increased fat cell size) and adipocyte hyperplasia (increased fat cell number) constitute the two fundamental mechanisms underpinning the storage capacity of adipose tissue. Adipogenesis, the process whereby precursor adipocytes differentiate into a diverse array of adipocytes, accumulate nutrients, and ultimately mature into adipocytes, is the primary reason for hyperplasia. Adipogenesis is a complex sequence of cellular differentiation processes, including the differentiation of mesenchymal stem cells into mesodermal precursor cells or germinal ganglion precursor cells, the differentiation of mesodermal precursor cells or myogenic ganglion precursor cells into various preadipocytes, and the differentiation of diverse preadipocytes into mature adipocytes (2). Moreover, adipose tissue consists of many types of cells, including endothelial cells, blood cells, fibroblasts, pericytes, precursor cells, adipocytes, macrophages and other immune cells (3, 4). Therefore, many scholars believe that fat is an important immune organ (5).

Adipose tissue is an active metabolic organ that necessitates multiple mechanisms to uphold its function and health. Autophagy represents one of these mechanisms. Autophagy is the process by which vesicles phagocytose intracellular material and fuse it with lysosomes to form autophagic lysosomes that degrade their contents, fulfilling the cell’s metabolic needs and renewing certain organelles. Most research has focused on the influence of autophagy on lipid metabolism, while fewer studies have examined the involvement of autophagy in adipogenesis. In recent years, evidence suggests that autophagy is a key regulator of WAT and BAT adipogenesis, and dysregulation of autophagy impairs fat metabolism (6). Many autophagy-related genes, autophagy-related pathways, autophagy-associated transcription factors and regulatory proteins participate in adipogenesis. Therefore, the modulation of autophagy presents a promising therapeutic avenue for treating obesity and its associated complications.

Adipocytes and immune cells within adipose tissue contribute to cytokine secretion, which plays indispensable roles in adipogenesis. These secreted cytokines can influence appetite regulation, energy metabolism, and immunological interactions (7). Immune cells within adipose tissue regulate various cytokines, and certain inflammatory factors, in turn, modulate adipogenesis (8). However, different inflammatory cytokines have different effects on adipogenesis. For example, TNF-α is often thought to function as an inhibitor of adipogenesis (8). However, the expression of IL6 and IL6R is positively correlated with adipogenesis differentiation of MSCs (9). The mechanisms by which inflammatory factors regulate adipogenesis and how dysregulated adipogenesis contributes to inflammation-mediated complications associated with metabolic disorders are currently research hot spots. Moreover, oxidative stress also significantly impacts adipogenesis and adipocyte hypertrophy (10). Elevated peroxide levels within adipocytes can trigger mitochondrial membrane damage, resulting in ATP blockade within the adipocyte and the generation of reactive oxygen species (ROS). These events precipitate morphological and functional alterations in adipocytes (11). Recent findings suggested that in addition to hormonal stimulation, ROS and free radicals might also modulate preadipocyte differentiation (12, 13). This review summarises the most recent investigations concerning the regulatory mechanisms of adipogenesis involving autophagy, oxidative stress, and inflammation. Most of the current studies have focused on the mechanism of single factor regulating adipogenesis, but there is still a need to incorporate multifactorial analyses. For example, autophagy has been playing a protective role by regulating inflammatory responses and ameliorating stress-induced cellular damage, thereby maintaining cellular homeostasis during stress. Therefore, we speculate that the interaction between oxidative stress, inflammatory factors and autophagy plays an important role in adipogenesis. Here, we discussed the emerging intersections between autophagy, oxidative stress, and inflammation in the context of adipogenesis for the first time. A comprehensive understanding of their roles will facilitate the identification of molecular targets for managing metabolic disorders associated with obesity.

## 2 The significance of autophagy in adipogenesis

Autophagy is an alternative cell death pathway within cells that involves the breakdown of damaged cellular components through targeted transport to specialized structures called lysosomes. This degradation process produces energy and replenishes metabolic reservoirs within the cell. Autophagy regulates cellular energy metabolism, maintains intracellular environmental balance, and supports metabolic renewal. Eukaryotic cells maintain the homeostasis of energy metabolism through the equilibrium of nutrient synthesis and degradation, encompassing proteins and lipids. The primary role of basal autophagy resides in the degradation of long-lived intracellular proteins and the elimination of damaged or senescent organelles. This intricate process plays a critical function in the preservation of cellular homeostasis. Within adipose tissue, there mainly exist macroautophagy/autophagy, mitophagy and lipophagy. These different forms of autophagy function in collaboration with one another during various physiological states. Their primary function is to govern the quantity, production, structure, and functioning of preadipocytes/adipocytes, thereby influencing adipogenesis. Most research has focused on the influence of autophagy on lipid metabolism, while fewer studies have examined the involvement of autophagy in adipogenesis. This section discusses the recent advancements in understanding the regulation of adipogenesis by autophagy and infers promising targets for manipulating adipogenesis.

### 2.1 Autophagy-related genes are key factors influencing adipogenesis

#### 2.1.1 ATG5 and ATG7

The discovery of autophagy-related genes (ATG) in yeast in 1990 provided powerful genetic and molecular tools for studying autophagy. More than 35 ATGs have been identified in yeast to date, and the 15 core ATGs required for starvation-induced autophagy are similarly highly conserved in mammals (14). ATGs play essential roles in all stages of autophagy. To understand whether autophagy affects other processes, we must first consider whether ATGs regulate these processes. Studies have shown the involvement of ATG in adipogenesis has been recognized. The initiation of embryonic fibroblast differentiation in mice lacking the ATG5 gene resulted in impaired adipogenesis and a substantial decline in the abundance of white adipocytes (15). This noteworthy finding underscores the involvement of ATG5 in the regular process of adipocyte differentiation, thus implicating a key role in autophagy during adipogenesis. AF4/FMR2 family member 4 (AFF4) is the scaffold protein of the super elongation complex, a class of transcriptional regulators involved in regulating cell development and differentiation (16). Studies have found that it plays an important role in differentiating various cells (17, 18). A recent investigation has demonstrated that the absence of AFF4 in hMSCs and 3T3-L1 preadipocytes inhibits cellular adipogenic differentiation. Over-expression of ATG5 and ATG16L1 rescues the impaired adipogenesis observed in cells where AFF4 is knocked down. Further studies have revealed that AFF4 can directly bind to autophagy-related proteins ATG5 and ATG16L1, thereby impacting adipogenesis (19). Similarly, mice with a deficiency in the ATG7 gene exhibited a remarkable suppression of autophagy, leading to diminished adipocyte differentiation and decreased accumulation of lipid droplets. The mutant mice contained only 20% of the mass of WAT found in WT mice. Crucially, the mutant mice did not appear to have any defects in other organs, and the weight of the lungs, kidneys, liver, heart, and brain did not differ significantly from that of the WT mice. Intriguingly, half of the mutant white adipocytes demonstrated multilocularity. Furthermore, the multilocularity observed in these mutant cells is not attributable to the increase in lipolysis, given that the mutant adipocytes displayed an unaltered basal lipolysis rate and even a reduction in hormone-stimulated lipolysis. These mice were found to be resistant to obesity resulting from a high-fat diet and demonstrated heightened insulin sensitivity (20, 21). This result is likely attributable to either increased energy expenditure, diminished energy utilization and storage efficiency, or both. Notably, the mutant mice exhibit pronounced hyperactivity. In the adipose-specific ATG7-KO models, there is a significant decline in plasma leptin concentrations, which is plausibly a consequence of the substantially decreased WAT mass. Furthermore, deleting the ATG7 gene in adipocytes decreased serum levels of free fatty acids and improved high-fat diet-induced steatosis, liver inflammation, and fibrosis (22). In general, mice models of lipoatrophy exhibit hyperlipidemia and insulin resistance due to lipid storage defects and abnormal lipid deposition in the liver and muscle. Intriguingly, mice with adipose-specific ATG7 knockout exhibit euglycemia and heightened insulin sensitivity. It is postulated that the morphological alterations within the mutant white adipocytes have precipitated fascinating functional modifications. In alignment with an elevated mitochondrial content, these mutant adipocytes display augmented rates of fatty acid β-oxidation. Furthermore, these mutant adipocytes demonstrate diminished rates of hormone-induced lipolysis. These alterations in lipid metabolism within the mutant WAT may have instigated the observed changes in lipid homeostasis within the mutant mice, characterized by reduced plasma levels of free fatty acids (FFA) during feeding and expedited rates of FFA reduction following insulin stimulation. Consequently, these systemic alterations in FFA homeostasis likely contribute to the amplified insulin sensitivity. In addition, ATG7 deficiency also affects brown fat differentiation (23). Recently, a study illustrated that the suppression of FTO (m6A demethylase) expression led to a decrease in the expression of ATG7 and ATG5, thereby hindering the formation of autophagosomes and impeding autophagy and adipogenesis. The FTO knockout mice exhibited a reduction in ATG5 and ATG7-dependent autophagy and a substantial decrease in white adipose tissue compared to the wild-type mice (24). These findings suggested that the knockdown of both ATG7 and ATG5 genes resulted in a decline in the differentiation of lipogenic cells and the accumulation of lipids.

#### 2.1.2 Other ATGs

**Figure 1:**
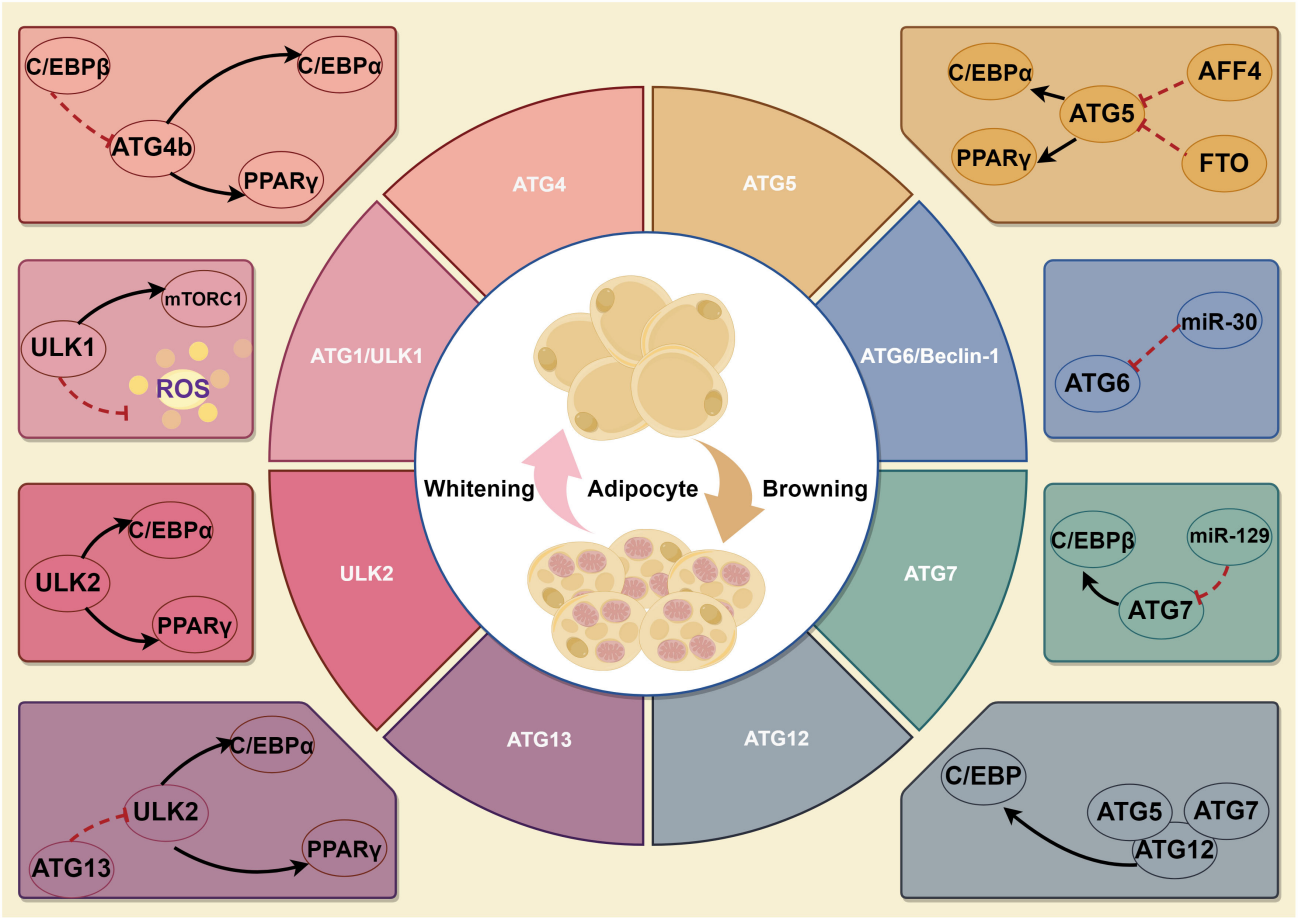
A schematic illustration depicting the role of ATGs in adipogenesis. Firstly, ULK1 is not required for adipogenesis, but ULK2 is indispensable for adipogenesis. Though ULK1 is dispensable for adipogenesis, it still affects autophagy, lipid metabolism and glucose uptake in adipocytes. Of which, YBX1 not only can promote ULK1/2-mediated autophagy during white adipogenesis but also induce brown adipogenesis via PINK1/PRKN-mediated mitophagy. Secondly, ATG4b can regulate PPARγ and C/EBPα, key positive adipogenesis regulators. Thirdly, ATG5 and ATG7 promote white adipogenesis and are involved in BAT whitening. In addition, ATG6 regulates autophagy in mature adipocytes, but whether it can affect adipogenesis remains unknown.

The autophagy initiation phase begins with activation of the ATG1 (the homologous gene in mammals is ULK1) complex. There are five ULK1 homologs in the human genome: ULK1, ULK2, ULK3, ULK4 and STK36. As an evolutionarily conserved autophagy regulation-associated serine/threonine protein kinase, ULK1 is responsible for ATG5- and ATG7-independent autophagy (25). The RNAi results revealed that ULK1 is not required for adipogenesis, but ULK2 is indispensable for adipogenesis in 3T3-L1 cells (26). The expression levels of PPARγ and C/EBPα, positive regulators of adipogenesis, were dramatically decreased in cells with knockdown of ULK2, whereas no such decrease was observed in ULK1-knockdown cells. Although ULK1 is dispensable for adipogenesis, inhibition of ULK1 would enhance insulin-responsive glucose uptake and lipid accumulation. On the other hand, ULK1 inhibition leads to increased oxidative stress, thereby potentially exacerbating the development of insulin resistance in adipocytes. ULK1 orchestrates lipid metabolism and facilitates glucose uptake in adipocytes, distinguishing itself from other ATGs. Notably, Y-box binding protein 1 (YBX1), an RNA binding protein, not only can promote ULK1/2-mediated autophagy during white adipogenesis but also can induce brown adipogenesis via PINK1/PRKN-mediated mitophagy (27, 28). With the deepening of research, many biochemical experiments have discovered that more ATGs may be involved in adipogenesis. Chromatin immunoprecipitation (ChIP) results suggested that C/EBPβ binding sites were found on the promoters of five autophagy-related genes-ATG2a, ATG4b, ATG7, ATG9a, and ATG10. However, among these genes, only ATG4b expression was significantly repressed by C/EBPβ siRNA, suggesting that ATG4b is transcriptionally regulated by C/EBPβ (29). Further study confirmed that ATG4b knockdown blocked the terminal differentiation of 3T3-L1 cells. ATG4b expression levels are highly regulated during adipogenesis, and transactivation of ATG4b by C/EBPβ is required to effectively activate autophagy and adipogenesis. Another study has reported that the down-regulation of Beclin-1 (Vps30/ATG6) expression inhibits autophagy in mature adipocytes and adipose tissue of mice following a high-fat diet. The consequences of high-fat diet-induced inhibition of autophagy include the following aspects: accumulation of damaged proteins and organelles, increased oxidative stress, impaired metabolic regulation, enhanced inflammation, development of insulin resistance, increased risk of neurodegenerative diseases, altered immune response, liver dysfunction and cardiovascular disease (30). This study found that in HFD-fed mice, there was an increase in the mRNA expression of several autophagy-related genes (Beclin-1, ATG5, ATG7, and ULK2) compared to chow-fed mice. However, the expression of Gabarapl1 did not significantly change. The protein level of LC3-II, an indicator of autophagy, was also higher in HFD-fed mice, suggesting increased autophagy in obesity. The study also found that berberine suppressed Beclin-1 mRNA expression and reduced LC3-II levels in HFD-fed mice, indicating that berberine could inhibit autophagy. In visceral fat, similar results were observed. This discovery highlights the critical involvement of Beclin-1 as a molecule in regulating autophagy in mature adipocytes (26, 31). But whether it affects adipogenesis remains to be further studied. Moreover, several studies suggest that ATG5、ATG7 and ATG12 participate in BAT whitening, expanding white adipogenesis (32, 33). Within adipogenesis research, it is necessary to focus on the factors that affect the expression and activation of core ATG proteins (**Figure 1**).

### 2.2 Autophagy-related pathways in adipogenesis

#### 2.2.1 mTOR is a crucial nod that links autophagy and adipogenesis

For a more comprehensive understanding of the relationship between autophagy and adipogenesis, we must also understand the role of autophagy-related pathways in this process. Several studies have demonstrated the interconnectedness of autophagy and adipogenesis through key signalling nodes, including mTOR, Notch, AMPK, and SIRT. TOR, as a key protein kinase signalling hub, senses changes in intra- and extracellular nutrient content, changes in energy levels, growth factors and other signalling stimuli, and regulates anabolic processes such as cell growth, proliferation, and protein synthesis through activation of its downstream effector proteins. In mammalian cells, mTOR is an important signal regulating autophagy, ribosome biosynthesis and protein synthesis and a key node in regulating signalling pathways related to nutrient metabolism *in vivo*. Activation of mTORC1, for instance, would influence various autophagy-related proteins and genes, such as Beclin-1 and LC3 I/II, thereby regulating autophagy. MTORC1 is susceptible to rapamycin, which is a rapid inhibitor of mTOC1. Treatment of hepatocytes with rapamycin enhanced intracellular autophagic activity and increased lipid oxidation levels and lipolytic activity (34). In C. elegans, rapamycin treatment enhances lysosomal acid lipase activity in cells (35). One recent study revealed that metformin upregulated Beclin-1 and LC3 I/II expression and downregulated mTOR expression, thereby stimulating autophagy induction. Also, metformin can mitigate adipogenesis of fibro-adipogenic progenitors after rotator cuff tears by mediating mTOR/ULK1-regulated autophagy (36). The inhibitory effect of metformin on mTOR may be mediated through AMPK. It has been shown that AMPK can inhibit mTORC1 in at least two parallel pathways: one is by phosphorylating TSC2 to directly inhibit the viability of RHEB and mTORC1, and the other is by directly phosphorylating the RAPTOR on the mTORC1 complex, which results in a structural change in the complex and thus inhibits the viability of the complex (37). It is known to all that the important biological functions of metformin are its attenuation of mitochondrial respiratory chain complex I activity and its enhancement of phosphorylation of AMPK (38). Under hyperglycemic conditions, enhanced autophagy is crucial in potentiating osteogenic differentiation in adipose-derived stem cells and ultimately mediates adipocyte differentiation (39). Alternatively, type I collagen has been shown to reduce intracellular lipid accumulation and adipocyte differentiation by inactivating autophagy through the YAP pathway. In the presence of collagen-coating conditions, there is an increase in lysosome-mTOR co-localization, accompanied by elevated downstream p-S6K protein expression and subsequent autophagy suppression (40).

#### 2.2.2 Notch signal in adipogenesis

The Notch signalling pathway, encompassing Notch, ligand Delta/Serrate/LAG-2 and CBF1/Su (H)/Lag-1 (CSL, a DNA-binding protein), is a critical component of cellular communication, orchestrating many cell differentiation processes (41). Notch and its ligands are single transmembrane proteins and play indispensable roles in adipogenic differentiation. The expression of PPARγ and C/EBPα was impeded upon exposure to the Notch ligand jagged1 or overexpression of the Notch target gene Hes-1 in 3T3-L1 cells. Intriguingly, these cells’ adipogenic differentiation potential was attenuated following Hes-1 knockdown via siRNA (42). A finding has elucidated that the inhibition of Notch signalling promotes autophagy-mediated adipogenic differentiation of MSCs through the PTEN-PI3K/AKT/mTOR pathway (43). In addition to its role in adipogenic differentiation, Notch signalling has been implicated in suppressing osteogenic differentiation via the inhibition of Wnt/β-catenin signalling (44). However, other studies have suggested that Notch signalling may promote osteogenic differentiation through interaction with BMP2 signalling (45). Consequently, the Notch signalling pathway modulates both adipogenesis and osteogenesis of MSCs through direct gene targeting or interaction with other signalling pathways. Interestingly, inhibiting autophagy through the inhibitor DAPT reduces Notch signalling activity, consequently impairing osteogenic differentiation in Dop ASCs. Nonetheless, the impaired osteogenic differentiation of Dop ASC can be restored by administering Torin1, a selective inhibitor of mTORC1/2 and autophagy agonist, which activates the Notch signalling pathway via the upregulation of autophagy (46).

#### 2.2.3 AMPK is a promising target for equilibrating autophagy and adipogenesis

AMPK, as a regulatory centre of cellular energy metabolism, can regulate individual metabolic emergencies by directly acting on metabolism-related proteins or indirectly affecting gene expression. Under stress, activated AMPK can directly regulate various downstream metabolism-related enzymes, such as mTOR, acetyl-CoA carboxylase, fatty acid synthase, glycerol phosphate acyltransferase, and PPARγ coactivator 1α (PGC-1α), to regulate different energy production/consumption pathways, thus maintaining the balance of cellular energy metabolism and homeostasis. AMPK is localized to the lysosome during autophagy, and AMPK regulates ULK1 complex activity by antagonizing mTORC1 and PI3KC3/VPS34 complex activity (47). Moreover, the mitochondria-localized AMPK is also involved in the regulation of mitophagy (48). The regulation of lipid metabolism is the primordial role of AMPK, whereby its activation manifests in a concomitant reduction in lipid storage (49). AMPK exerts a suppressive influence on the *de novo* biosynthesis of cholesterol, triglycerides, and fatty acids. It both inhibits and phosphorylates substrates intricately associated with the biosynthesis of fatty acids. AMPK impairs cholesterol biosynthesis by phosphorylating and suppressing the enzymatic activity of 3-hydroxy-3-methylglutaryl-coenzyme A (HMG-CoA) reductase (50). Furthermore, AMPK elicits the augmentation of mitochondrial genesis and β-oxidation utilizing the orchestration of PGC-1α activity (51). The loss of PGC-1α functionality precipitates a diminished manifestation of mitochondrial and thermogenic genes within white adipose tissue. AMPK has been demonstrated to exert an inhibitory effect on adipogenesis by impeding the initial stages of mitotic clonal expansion, concomitant with diminished expression of early and late adipogenic factors, including fatty acid synthase, sterol regulatory element-binding protein-1c, and adipocyte protein 2 (52). The suppression of adipogenesis through the utilization of small-molecule activators (RSVA314 and RSVA405) targeting AMPK, with concomitant inhibition of the MCE phase, accompanied by decreased expression of C/EBPβ, suppression of C/EBPα, PPARγ, and the subsequent downregulation of late adipogenic factors, including SREBP-1c, FAS, and FABP4, was observed (53). Similarly, the activation of AMPK by A769662 led to a decrease in lipid droplets and the activation of PPARγ, C/EBPα, as well as early adipogenic transcription factors such as C/EBPβ and C/EBPδ (54). A study suggests that the effect of AMPK on adipogenesis is autophagy-dependent. Pre-inhibiting or pre-promoting autophagy with siATG7 or rapamycin would block AMPK. The study showed inhibiting or improving autophagy would promote or inhibit the role of AMPK prohibition in adipose-derived stem cell adipogenesis (55). Moreover, the selection of AMPK-mediated signalling cascades also involved BAT activation. These pathways are categorized into three distinct processes: the development of brown adipocytes, mitochondrial health, and browning. Firstly, the AMPK/mTOR axis is the core signal involved in autophagy and protein synthesis in the developmental stage and glucose uptake in adult brown adipocytes. Secondly, AMPK can activate PRDM16, an important DNA-binding transcription factor that indirectly induces adipogenesis and lipolysis, via α-ketoglutarate. Thirdly, AMPK triggers the activation of SIRT1 via deacetylation, culminating in the activation of the peroxisome PGC1α and subsequent augmentation of mtDNA content, mitochondrial dimensions, and abundance, ultimately resulting in diminished adipogenesis. Finally, AMPK affects the abundance of UCP1 expression, which is a key factor in WAT browning (56). These compelling evidences indicate that AMPK plays a pivotal role in developmental and functional processes of BAT. It also has been showed that AMPK has the capacity to elicit BAT activation through a diverse array of signaling pathways. Meanwhile AMPK-mediated autophagy and mitophagy play important roles in this process. Clarifying the roles of autophagy related signaling pathways in adipogenesis is helpful to explore molecular targets for the treatment of obesity-related metabolic disorders. Next we will discuss the specific nodes where these factors or pathways act on adipogenesis.

### 2.3 Autophagy-associated transcription factors and regulatory proteins regulate adipogenesis

Additional investigations have revealed that other autophagy-associated proteins, regulatory factors, and transcription factors exert direct or indirect influences on the process of adipogenesis. Some adipogenic factors regulate adipogenesis by targeting autophagy-associated genes (such as Beclin-1, LC3B, and p62) and autophagy transcription factors (including FOXO1, TFEB, and XBP1) (57). In bone marrow-derived mesenchymal stem cells (BMSCs) expressing Scara3, p62 was suppressed while the expression of LC3B and FOXO1 was upregulated, thereby promoting autophagy (58). Oil Red O staining results demonstrated that overexpression of Scara3 impeded lipid droplet formation. However, this effect was disrupted by silencing FOXO1b expression. Therefore, Scara3 potentially affects lipid differentiation through the FOXO1-autophagy signalling axis. Recently, it has been determined that microRNA (miRNA) and long non-coding RNA (lncRNA) play pivotal roles in autophagy and adipogenesis. For example, miRNA-9 targets PNPLA3, reducing lipid droplet accumulation and triglyceride content while promoting AMPK pathway phosphorylation, ultimately inhibiting adipogenesis (59). Bioinformatic analysis has predicted that the lncRNA, lncIMF4, is involved in various biological processes, including cell proliferation, differentiation, and autophagy. Knockdown of the lncIMF4 gene in pigs has resulted in a significant upregulation of the autophagy-associated gene p62, downregulation of LC3, and a substantial increase in intracellular lipid droplets. These findings suggest that the knockdown of lncIMF4 attenuates lipolysis by suppressing autophagy, ultimately promoting intramuscular lipogenesis (60). Furthermore, it has been observed that the Vangl2 protein directly binds to lysosome-associated membrane protein and targets lysosomes for degradation. This process inhibits adipogenesis by scavenging lipogenic transcription factors TLE3 and ZNF423 (61). Deficiency of the transcription factor Nrf2 attenuates autophagic flux and inhibits the fusion of autophagosomes and lysosomes *in vivo* and *in vitro*. Subsequently, this decrease in autophagy leads to hepatic lipid accumulation. Additionally, adipogenesis can be stimulated by enhancing the activity of the transcription factor SREBP-1c (62). Collectively, the studies mentioned above indicate that autophagy-associated transcription factors and regulatory proteins possess the potential to regulate or interact with factors implicated in adipogenesis and related pathways (**Table 1**). Thus, the modulation of autophagy is a promising strategy for preventing and controlling obesity through influencing adipogenesis.

**Table 1:** Autophagy-associated factors regulate adipogenesis.

Autophagy-related gene	Effect on adipogenesis	References
ATG5/ATG7	FTO promotes the expression of ATG5/ATG7 and induces autophagosome, affecting adipogenesis	(24)
ATG5/ATG16L1	AFF4 directly binds to ATG5 and ATG16L1 and regulates autophagy during adipogenesis	(19)
Rubicon	Loss of RUBCN mediates the upregulation of autophagy, which further causes a reduction in NCOA1/2 level, inhibiting adipogenesis.	(63)
Pink1/Parkin	Loss of YBX1 decreases the Pink1/Parkin level, alleviating mitophagy and inhibiting the thermogenic program.	(28)
Type I collagen	Type I collagen inactivates autophagy by up-regulating YAP-mediates mTOR activity.	(40)
Scara3	Scara3 controls the cell fate by promoting Foxo1 expression and autophagy flux.	(58)
Nrf2	Nrf2 deficiency attenuates autophagic flux and inhibits the fusion of autophagosomes and lysosomes.	(62)
PNPLA3	PNPLA3 plays an essential role in lipophagy in hepatocytes	(59)
lncIMF4	knockdown lncIMF4 facilitates intramuscular adipogenesis through attenuating autophagy to repress the lipolysis	(60)
FUNDC1	Ablation of FUNDC1 results in defective mitophagy and impaired mitochondrial QC	(64)
TP53INP2	Autophagy-related protein TP53INP2 activates autophagy during the early stage of differentiation in bovine adipocytes and positively regulates adipocyte differentiation by affecting autophagy.	(65)

### 2.4 Autophagy regulates key adipogenesis regulators PPARγ and C/EBPα

On the basis of summarizing promising autophagy-related genes and pathways affecting adipogenesis, we still hope to identify the specific targets of autophagy regulation of adipogenesis. Therefore, we focus on some adipogenesis regulators. PPARγ plays a pivotal role in regulating adipocyte differentiation, while TP53INP2, an autophagy protein, stimulates autophagy and promotes PPARγ expression. The activation of PPARγ effectively compensates for the decline in lipid droplets caused by TP53INP2 knockdown, thus governing the adipocyte differentiation process (65). Fasting induces a notable decrease in Rubicon, a negative regulator of autophagy, in adipose tissue, which is accompanied by an increased level of autophagy. Adipose-specific Rubicon-knockout mice exhibit systemic fat loss (63). Implied in the autophagy-induced reduction of adipogenic gene expression is the degradation of coactivators of PPARG/PPARγ, specifically NCOA1/SRC-1 and NCOA2/TIF2. The degradation of these substrates during fasting leads to a decline in mRNA levels of adipogenic genes in adipocytes. Furthermore, the knockout of Rubicon in adipocytes results in adipose atrophy in the liver, owing to the downregulation of adipogenic gene expression. The activation of PPARγ can restore the expression of these genes. Additionally, autophagy facilitates the degradation of SRC-1 and TIF2, coactivators of PPARγ. This degradation relies on their interaction with GABARAP family proteins and is significantly reduced in Rubicon-deficient or senescent adipocytes (66). Mechanistically, the excessive autophagy instigated by Rubicon inhibition within adipocytes precipitates an LIR/GIM-dependent diminution of SRC-1 and TIF2, functioning as coactivators of PPARγ. It is postulated that SRC-1 and TIF2 within adipocytes are translocated to the cytosol and degraded via autophagy in response to external energy demand. These reductions instigate a decline in PPARγ activity and adipocyte function, facilitating energy supply for other tissues. Moreover, given that the overexpression of LIR/GIM mutant SRC-1 and TIF2 failed to fully rescue the reduction in adipogenic gene expressions instigated by Rubicon knockdown, it is plausible that the absence of Rubicon fosters the autophagic degradation of other specific constituents implicated in adipogenesis. C/EBPα overexpression upregulates the expression of LC3B, ATG5 and Beclin1 and further induces autophagy. Moreover, C/EBPα affects the expression of P62 and its binding to Beclin1 through acetylation modification (at positions K298, K302 and K326) to induce autophagy (67). In turn, pre-promoting or pre-inhibiting autophagy with rapamycin or 3MA decreased or increased C/EBP-α expression, but the underlying mechanism remains unknown (68). At present, there are few clear targets for autophagy to regulate adipogenesis. Exploring these targets and analyzing their mechanism will be hot topics for the next research.

### 2.5 Mitophagy has effects on fat metabolism and adipogenesis

While the above discussion mainly focused on the effect of macroautophagy (autophagy) on adipogenesis, this section will specifically discuss the role of selective autophagy in adipogenesis, which is the current research hotspot. Several recent studies suggest that selective autophagy (especially mitophagy) may be a key factor influencing adipogenesis (28, 69, 70). The process of adipogenesis is the result of intracellular energy changes and metabolism. Mitochondria, as a vital biological energy centre, must be paid more attention. Mitophagy is the targeted phagocytosis and destruction of mitochondria by cells and is generally regarded as the main mechanism of mitochondrial quality control. Initiation of mitophagy involves the recruitment of the cytosolic E3 ubiquitin ligase PARK2/Parkin to the injured mitochondrion, facilitated by the protein kinase PINK1. Once they are recruited, PARK2 ubiquitinates mitochondrial substrates, thereby initiating mitophagy. This intricate process assumes a central role in cell types rich in mitochondria, such as brown and beige adipocytes, where it governs adipocyte differentiation and supports homeostasis within beige adipocytes (71). Interestingly, the mitophagy receptor FUNDC1 dysfunction significantly affects adipose inflammatory metabolism and insulin resistance, compromising adipocyte production and exacerbating diet-induced obesity (64). Mechanistically, the hyperactivation of the MAPK/JNK pathway precipitates insulin resistance, a condition ameliorable through the abrogation of MAPK8/JNK1 in the FUNDC1 knockout model. Dysregulated maintenance of mitochondrial quality control, stemming from impaired mitophagy receptor FUNDC1, establishes a connection with fat metabolism disorders through the mediation of MAPK signalling and the orchestration of inflammatory responses. As a newly identified mitophagy receptor, Bcl-2-like protein 13 (Bcl2l13) has been demonstrated to be critical for adipogenic differentiation (72). Bcl2l13 knockdown significantly impaired adipocyte differentiation. Knockdown of Bcl2l13 triggered cellular reprogramming, augmenting the reliance on glycolysis to fulfil ATP requisites in compromised oxidative phosphorylation. Bcl2l13 depletion in embryonic mesenchymal stem cells induced increased mitophagy. Furthermore, Bcl2l13 also served to alleviate apoptosis during the process of adipogenesis. As one of the best-studied selective autophagy receptors, p62 takes part in different types of selective autophagy, including aggrephagy, pexophagy, and mitophagy. The p62 is an adapter molecule, engaging directly with ubiquitinated molecules on the autophagosome. The inhibition of p62 entirely obstructs the clearance of compromised mitochondria. Hence, activating the PINK1/PARKIN/p62 axis is important in effectively eliminating damaged mitochondria, a process indispensable for maintaining their quality control. P62 has been shown to play an important role in adipose tissue metabolism and adipogenesis. Obesity and insulin resistance have been noted in p62-deficient murine models, with basal lipolytic hydrolysis manifesting a decreasing tendency compared to their wild-type counterparts. Furthermore, p62-knockout mice exhibit an augmented quantity of intracellular lipid droplets, elevated triglyceride synthesis, and an enlargement in adipocyte size (73). Specifically, the main organ affected is the BAT, where p62 controls mitochondrial function directly. The deficiency of p62 in BAT affects mitochondrial structure and function and impairs thermogenesis. The study also shows that p62 controls the activation of p38 *in vivo*, which is crucial for BAT nonshivering thermogenesis and UCP1 function. The loss of p62 in adipocytes impairs the activation of p38 and its downstream pathways, affecting uncoupling *in vivo* and *in vitro*. It suggests that p62 controls mitochondrial function via the p38/Ppargc1a pathway. The study also found that oxidative capacity, measured by Cox activity, was decreased in BAT of adipocyte-specific p62-knockout mice. The study concludes that p62 plays a critical role in regulating transcriptional programs controlling mitochondrial homeostasis, which underlies the phenotype of adipose-specific p62-KO mice. A recent study showed that p62 knockdown enhanced mitophagy (74). Knockdown of p62 in human adipose-derived stem cells (hADSCs) yielded a pronounced decrement in stabilizing capacity, consequently culminating in heightened mitophagy. Conversely, suppressing p62 increased the efficiency in activating alternative mediators, thus instigating mitophagy. In this case, the role of p62 as a mediator for mitophagy becomes dispensable. During the adipogenic differentiation of hADSCs, the knockdown of p62 led to an uneven distribution of autophagic fluxes: a relatively increased mitophagy flux. Consequently, the refreshment of mitochondria was ensured, further propelling the synthesis of lipids and the progression of differentiation. Notably, YBX1, an RNA-binding protein, exhibits robust expression in BAT and is induced by exposure to cold and β-adrenergic agonists in mice. Loss-of-function experiments have illustrated that YBX1 deficiency impairs the differentiation of primary brown adipocytes and their capacity for thermogenesis. Subsequent investigations have revealed that YBX1 exerts a positive regulatory influence on thermogenesis by enhancing mitophagy (28). RNA immunoprecipitation studies have identified direct targeting of PINK1 and PARKIN transcripts by YBX1. Additionally, RNA decay assays have demonstrated that YBX1 deficiency diminishes the mRNA stability of PINK1 and PARKIN, leading to reduced protein expression. Consequently, this impairment compromises mitophagy and represses the thermogenic program. At present, there are few studies on the regulation of adipogenesis by mitophagy. We believe that further research in this area will help us understand the changes in energy metabolism and the mechanism of cell transformation (the differentiation of diverse preadipocytes into mature adipocytes).

## 3 New advances in the impact of oxidative stress on adipogenesis

### 3.1 Oxidative stress is an important driver of adipogenesis

In addition to autophagy, oxidative stress is closely related to intracellular material and energy metabolism. So, we’re also interested in its role in adipogenesis. Oxidative stress refers to the discrepancy between the generation of ROS in the cell’s internal and external environment and the cell’s intrinsic antioxidant capacity, resulting in a disturbance of the intracellular redox balance. This imbalance in a tendency to oxidize impairs normal cellular function.

Oxidative stress is strongly associated with adipogenesis, as heightened levels of peroxides within adipocytes cause damage to the mitochondrial membrane, hindering ATP production and generating ROS. In turn, it contributes to morphological and functional alterations in adipocytes. Generally, adipocytes exhibit a higher spontaneous production of intracellular and extracellular ROS than preadipocytes (75). For instance, the expression of MCPIP induces the production of reactive oxygen species/reactive nitrogen species in 3T3-L1 cells and enhances adipogenesis. Conversely, inhibiting the expression of reactive oxygen species hampers the ability of MCPIP to stimulate adipogenesis (76). Furthermore, oxidative stress induced by heme inhibits Sirt1, which disrupts the regulation of PPARγ and C/EBPα. These factors are known to promote adipogenesis and preadipocyte hypertrophy (77). NOX4, a crucial NADPH oxidase, belongs to a protein class that converts oxygen into reactive oxygen radicals. Catalase-knockout mouse embryonic fibroblasts appeared to differentiate into adipocytes more easily than wild-type cells. Silencing catalase significantly increases H_2_O_2_ concentration, followed by increased NOX4 expression and decreased AMPK levels. Ultimately, this cascade leads to adipogenesis (78). Suppression of the adipose-specific protein BAMBI results in increased expression of NOX4. The study found that increased white fat deposits in BAMBI knockout mice were due to elevated levels of NOX4. NOX4 is involved in stimulating ROS production in mitochondria and C/EBPβ expression. Another study also shows that administering the antioxidant NAC to BAMBI knockout adipocytes can reverse lipogenic differentiation (79). MSCs are adult stem cells with self-renewal capacity and multidirectional differentiation potential, and the expression level of cystathionine β-synthase in human adipose MSCs is low. This low level is accompanied by increased inflammatory factors and markers of oxidative stress, including elevated intracellular reactive oxygen species and decreased intracellular glutathione levels. They lead to an upregulation of cellular lipogenic genes (80). In glucocorticoid-induced osteoporosis, MSCs display a substantial reduction in the expression of osteogenic genes and an increase in lipogenic gene expression. It is due to GCs elevating the level of oxidative stress in the cells and inducing the expression of SENP3, promoting lipogenic differentiation. Further studies have demonstrated that SENP3 knockdown suppressed the detrimental effects of glucocorticoid-induced osteoporosis on osteogenic differentiation and reduced lipid formation and triglyceride content. These findings suggest that SENP3 knockdown inhibits lipid accumulation and lipogenic differentiation through oxidative stress (81). GPX7, an antioxidant enzyme located on the endoplasmic reticulum, inhibits ROS production and promotes osteogenic differentiation of bone marrow MSCs by stimulating endoplasmic reticulum stress and the mTOR pathway. Knockdown of GPX7 in MSCs leads to an increase in lipogenic capacity (82).

Although the prevailing view is that oxidative stress contributes to adipogenesis, some studies still report that oxidative stress may impede adipogenesis under certain circumstances. Suppression of protein tyrosine phosphatase 1B (PTP1B) enhances mitochondrial dynamics, attenuates oxidative stress, and potentiates the adipogenic differentiation capacity of adipose-derived stem cells (83). In a comparable investigation, the activity of PTP1B was obstructed by MIS-1436, resulting in diminished oxidative stress, decreased expression of endoplasmic reticulum stress-related proteins, and heightened levels of apoptosis-related proteins. The cumulative effect of these mechanisms may have synergistically amplified the adipogenic differentiation potential of cells towards mature adipocytes (84). Prolonged exposure of adipocytes to low levels of H_2_O_2_ elicits changes in mitochondrial dynamics, reduces the efficiency of cellular respiration, and down-regulates the expressions of PPARγ and C/EBPα, as well as the lipogenic marker Plin1. Ultimately, these events culminate in impaired adipogenesis (12). Moreover, there is compelling evidence that ROS can impede adipogenesis by up-regulating the lipogenic inhibitory factor CHOP-10/GADD153 (85). It suggests that oxidative stress may play a double-edged role in regulating adipogenesis. These opposite results may be due to differences in the intensity and timing of oxidative stress or to a combination of other factors. Therefore, more studies are needed to uncover the special mechanisms involved.

### 3.2 Oxidative stress regulates energy metabolism in BAT

Although most of the research on adipogenesis has been done in WAT, the growth and differentiation of brown adipocytes have also attracted much attention from researchers. A recent study has uncovered that the intertissued administration of enoxacin remarkably augments the mitochondrial count within adipocytes, reinforcing fatty acid oxidation metabolism and fuel consumption while diminishing adipocyte size and body fat composition (86). Enoxacin works by regulating miRNAs within adipose tissue, such as miRNA-34a-5p. Subsequently, oxidative metabolism is stimulated via the FGF21 signalling pathway, and the expression of thermogenic genes UCP1, Dio2, and Ppargc1a are activated. Ultimately, it promotes energy expenditure and decreases lipid accumulation. In a distinct investigation, it was observed that oxidative stress downregulates the expression of pivotal adipogenesis regulators, including PPARγ, C/EBPβ, and cell death-inducing DFFA-like effector A and FABP4, in mature brown adipocytes (87). As a result, adipogenesis in brown fat is impaired. Simultaneously, oxidative stress leads to a decline in the expression of thermogenic genes such as UCP1 and PGC-1α, thereby reducing thermogenesis and energy expenditure. While the effects of oxidative stress on adipogenesis remain controversial, researchers generally believe that oxidative stress can promote adipogenesis (**Table 2**). Differences in these findings may be due to different types of adipose tissue. The differential regulation mechanisms of adipogenesis by oxidative stress in WAT and BAT need to be further revealed. A comprehensive understanding of the role played by oxidative stress in adipogenesis would be pivotal in advancing our comprehension of the pathogenesis of obesity and associated disorders and facilitating the development of more productive prevention and treatment strategies.

**Table 2:** Oxidative stress-related factors regulate adipogenesis.

Target	Function	Effect on adipogenesis	References
Nrf2	Oxidative stress promotes Nrf2 recruitment to the sterol regulatory element binding protein one promoter, inducing lipogenesis.	Promoting	(13)
PPARγ	Sustained low levels of oxidative stress reduce the PPARγ level, inhibiting preadipocytes from differentiating to mature adipocytes.	Promoting	(12)
SENP3	SENP3 promotes adipose differentiation during oxidative stress by PPARγ2 DeSUMOylation	Promoting	(81)
MCPIP	MCPIP induces adipocytes to produce ROS/RNS, which in turn regulates key transcription factors for adipogenesis	Promoting	(76)
NOX4	NOX4 induces oxidative stress and reduces AMPK activity, resulting in disturbed energy metabolism and fat deposition	Promoting	(78)
CHOP-10/ GADD153	CHOP-10/GADD153 triggers hypoxia-dependent inhibition of adipocyte differentiation	Inhibiting	(85)
PTP1B	Inhibition of PTP1B reduces oxidative stress, improves mitochondrial biocompetence and kinetics, and enhances the lipogenic differentiation potential of adipocytes.	Inhibiting	(84)
Sirt1	Heme-dependent oxidative stress negatively regulates Sirt1 activity by enhancing the expression of lncRNA and subsequent adipogenesis.	Inhibiting	(77)
BAMBI	BAMBI inhibits NOX4 activity and reduces ROS expression, which in turn affects C/EBPβ activity, reducing lipid synthesis	Inhibiting	(79)
miRNA-34a-5p	MiRNA-34a-5p promotes energy expenditure and reduces lipid accumulation by mediating the FGF21 to induce oxidative metabolism.	Inhibiting	(86)

## 4 New revelations regarding the impact of inflammatory cytokines on the process of adipogenesis

Adipose tissue consists of many types of cells, including endothelial cells, blood cells, fibroblasts, pericytes, precursor cells, adipocytes, macrophages and other immune cells (3, 4). Consequently, adipose tissue is widely acknowledged as a primary reservoir of immune cells (5). Studies have revealed that immune cells play an intricate role in the secretion of inflammatory factors, which concurrently modulate adipogenesis. Although the impacts of certain inflammatory cytokines on adipogenesis have been reported, ongoing investigations are dedicated to discerning novel functions and roles of these cytokines in this physiological process. Recently, several other inflammatory factors have been found to impact adipogenesis.

### 4.1 TNF-α inhibits adipogenesis

TNF-α has demonstrated formidable suppressive effects on adipogenesis, primarily utilizing TNFR1 activation, leading to subsequent stimulation of the NF-κB, ERK1/2, and JNK signalling pathways. The reinstatement of 3T3-L1 cell differentiation can be achieved by impeding NF-κB and JNK signalling through specific inhibitors. In addition, the Wnt/β-catenin/TCF-dependent cascade and the repression of transcription factors are also involved in TNF-α-mediated inhibition of adipogenesis (8). A recent investigation has revealed that lactic acid bacteria-fermented skimmed milk exerts inhibitory effects on adipogenesis by hindering the activities of the principal transcription factor PPARγ (88). This effect is realized via the upregulation of the proinflammatory cytokine TNF-α in 3T3-L1 cells.

### 4.2 Interleukin-induced adipogenesis

IL-1β, an interleukin, is predominantly synthesized by THP-1 macrophages in adipose tissue and, to a lesser degree, by adipocytes. It impedes adipocyte production by binding to the IL-1β receptor and activating the NF-κB signalling pathway. IL-6, belonging to the GP130 family of cytokines, predominantly binds to the GP130 receptor to form homodimers or heterodimers, thereby facilitating signal transduction. In most instances, IL-6 is bound to the membrane IL-6Rα receptor, which induces β-receptor dimerization, activates the JAK/STAT signalling pathway, and transmits the signal intracellularly, thereby promoting inflammation (89). A small portion of IL-6 can also bind to soluble IL-6Rα, forming a complex that promotes energy metabolism through transmembrane signalling. Generally, the pro-inflammatory factor IL-6 regulates adipocyte production and counteracts obesity, but its role in insulin sensitivity remains controversial. Reduction of TNF-α and IL-6 downregulates the mRNA expression of PPAR-γ, sterol regulatory element binding protein-1c, and leptin. However, these cytokines lead to upregulation of lipocalin and uncoupling protein-1 (UCP-1) expressions (90). During Brucella infection, there is an upregulation of IL-6 and MMPs-2/9 secretion in preadipocytes and adipocytes, resulting in the downregulation of lipocalin and leptin expression in differentiated adipocytes (91). Moreover, it has been demonstrated that IL-6R promotes the adipogenic differentiation of MSCs by activating the p38 signalling pathway, as evidenced by both IL-6 receptor knockdown and overexpression experiments (9). IL-11, also a member of the Gp130 cytokine family, primarily targets adipocytes through the IL-11 receptor. It suppresses the mRNA expression of PPARγ and C/EBPα by inhibiting Dkk1 and Dkk2, thus augmenting Wnt signalling and impeding adipogenesis (92).

Macrophages, dendritic cells, cardiomyocytes, and smooth muscle cells predominantly secrete IL-20. The IL-20 receptor comprises a heterodimeric complex composed of IL-20Rα and IL-20Rβ subunits. Activation of IL-20 receptors triggers the activation of various signalling pathways, including STAT3, p38, and JNK, with STAT3 serving as the principal signal. During the adipose differentiation process, IL-20 induces the expression of TNF-α, which subsequently modulates adipose differentiation. In murine *in vitro* experiments, IL-20 has been shown to regulate the differentiation of adipocytes and the polarization of bone marrow-derived macrophages toward M1-type pro-inflammatory cells (93). Furthermore, IL-20 enhances the expression of netrin 1, leptin, and MCP-1 in adipocytes by upregulating TNF-α, MCP1, netrin 1, and un5b expression in macrophages, thereby promoting adipose tissue inflammation and macrophage retention. IL-17, predominantly produced by γδT cells, orchestrates a diverse repertoire of immune responses and primarily controls the initiation of pro-inflammatory reactions (94). In the context of adipocyte differentiation, IL-17 stimulates the production of PGE2 in human bone marrow mesenchymal stem cells (hBM-MSCs), thereby inhibiting the differentiation of preadipocytes. Additionally, IL-17 modulates adipogenesis by influencing the expression of Kruppel-like family members (KLF), including KLF15, KLF2, and KLF3, while also impeding the activity of PPARγ and C/EBPα. Notably, IL-17A markedly suppresses FABP4 and PPARγ and facilitates the recruitment of type 17 lymphocytes that harm adipose tissue function (95).

Additionally, some other interleukins also linked to inflammation are associated with adiposity. IL-38, an anti-inflammatory cytokine belonging to the IL-1 family, elevates the expression of GATA-3 and GLUT4 mRNA while suppressing the secretion of IL-1β, IL-6, and MCP-1 from 3T3-L1 cells (96). In turn, it restrains the differentiation of human adipocytes and the production of inflammatory cytokines by 3T3-L1 cells. In adipocytes, the knockdown of IL-21R diminished the lipogenic capacity of ADSCs without impacting the proliferation and mitochondrial activity of ADSCs. The relationship between IL-21 and the process of lipogenic differentiation warrants further investigation (97). IL-35 could regulate the equilibrium between osteogenic and lipogenic differentiation of progenitor cells through the Wnt/β-catenin-PPARγ signalling pathway. Moreover, IL-35 administration promotes osteogenesis while inhibiting adipogenesis (98). The effect of interleukins on adipogenesis deserves further study.

### 4.3 Regulation of adipogenesis by interferons

IFN-α is a polymorphic immunomodulatory cytokine produced by monocytes/macrophages, lymphoblasts, and various cell types in response to diverse stimuli. It is extensively studied for its role in treating cancer and viral infections. During adipocyte differentiation, IFN-α hinders adipocyte differentiation at the initial stages of adipogenesis by governing the expression of PPARγ and C/EBPα and regulating the cell cycle through modulation of the JAK/STAT1 signalling pathway (99). IFN-γ is a pro-inflammatory factor that serves various biological functions in immune regulation, tumour resistance, and induction of cell differentiation. In adipocytes, IFN-γ represses the expression of PPARγ, C/EBPβ, and C/EBPα while also triggering apoptosis in adipocytes. Studies have also shown that activated IFN-γ and CD8+ T cells promote lipogenesis in mice’s bone marrow mesenchymal stromal cells with aplastic anaemia *in vitro* or *in vivo* (100). Moreover, evidence suggests the interferon signalling pathway plays a regulatory role in lipogenesis (101). In mouse preadipocytes lacking IRF3, a heightened PPARγ and PPARγ-mediated lipogenic gene expression led to enhanced adipogenesis. Furthermore, another study has validated the significance of single nucleotide polymorphisms in the porcine IFN-α-16 and TNFRSF19 genes in promoting intramuscular fat deposition in pigs (102).

During the adipose differentiation and production process, there is a discernible alteration in the transcript levels of diverse miRNAs, and several miRNAs are intricately associated with inflammatory responses. Compare people with significant differences in obesity degree, a total of 25 miRNAs have been identified as targeting three upregulated adipogenesis-associated inflammatory genes, namely interleukin-6 (IL-6), tumour necrosis factor-alpha (TNF-α), and interleukin-1 beta (IL-1β). Remarkably, these miRNAs were observed to undergo conserved changes throughout adipogenesis (103). Furthermore, analysis of the relationship between TNF-α and miR-424 in childhood obesity has revealed that miR-424 is regulated by TNF-α and plays a crucial role in adipogenesis, with its mechanism of action being closely related to the Wnt/β signalling pathway (104). Continual exploration of the influence of inflammation on adipogenesis holds the potential to identify novel targets for ameliorating aberrant adipose tissue development. These invaluable findings may pave the way for developing novel therapeutic strategies to address obesity and its associated pathologies.

## 5 The crosstalk between autophagy, oxidative stress and inflammation plays a key role in regulating adipogenesis

The studies above suggest that several factors, including autophagy, oxidative stress, and inflammation, always influence adipogenesis outcomes. Although our studies predominantly focus on a single aspect of the adipogenesis regulatory mechanism, it remains necessary to incorporate multifactorial analyses. Autophagy consistently exerts a protective influence by regulating the inflammatory response and ameliorating stress-induced cellular damage, thus preserving cellular homeostasis during stresses. There exists a closed correlation between oxidative stress, inflammatory factors and autophagy. ROS can induce autophagy by directly modulating the activity of multiple upstream autophagy pathways, including AMPK, mTOR, MAPK, and PI3K. Appropriate autophagy can also suppress excessive inflammatory responses. Many pathologically relevant investigations have demonstrated the interplay between autophagy, oxidative stress, and inflammation, ultimately impacting the physiological state of tissues and organs (105–107). Although few studies have examined their roles in adipogenesis simultaneously, the synergistic and antagonistic effects of all three of them in adipogenesis may be of great significance. Here, we discussed the interplay between autophagy, oxidative stress, and inflammation during adipogenesis for the first time.

### 5.1 The autophagy-oxidative stress axis mediates adipogenesis

Firstly, there is a close link between oxidative stress and autophagy. ROS can initiate autophagy by directly modulating the activity of multiple upstream autophagy pathways, such as AMPK, mTOR, MAPK, and PI3K (108). ROS can also modify autophagy-related proteins, thereby regulating autophagy activity (109). Conversely, autophagy attenuates oxidative stress by eliminating damaged organelles and excessive oxidizing intermediates. Deficiencies in autophagy-related proteins contribute to the accumulation of cellular ROS. Recent studies have revealed both autophagy and oxidative stress are involved in adipogenesis. In the fibroblasts of patients with Graves’ orbitopathy, autophagy induced by IL-13 promotes inflammation, ROS production, and fibrosis, thereby impacting adipose differentiation. Conversely, neferine inhibits autophagy-associated inflammation, oxidative stress, and adipogenesis by activating Nrf2 and PI3K/Akt/mTOR signalling pathways (110). The mineralocorticoid receptor (MR) signalling pathway plays a key role in the normal physiological differentiation and maturation of adipocytes. However, excessive MR activation can lead to excessive oxidative stress, the release of pro-inflammatory cytokines, and dysregulation of adipocyte autophagy (111). In adipocytes, the MR antagonist suppresses the transcription of lipogenic and inflammatory cytokines by activating the Akt-FOXO1 pathway and reduces the transcriptional levels of ROS-producing enzymes, consequently promoting the transcription of adipose regulators PPARγ and sgk1, as well as autophagic flux (112). Overexpression of MCPIP in 3T3-L1 preadipocytes increases ROS production through p38 activation, which accordingly impacts autophagy levels and adipogenesis. Induction of precursor adipocytes using a combined approach enhances intracellular autophagy levels, but this effect is mitigated by MCPIP knockdown (76). Autophagy is activated in MSCs and plays a crucial role in their self-renewal and survival. Notably, adipose tissue-derived MSCs exhibit a superior phenotype to bone marrow-derived MSCs, as they possess a higher potential for proliferation and differentiation and a slower senescence rate (113). The hypoxic state of MSCs promotes the maintenance of their stemness, and hypoxia can trigger autophagy in MSCs. Furthermore, autophagy in MSCs is regulated by ROS. Thus, in MSCs, the intracellular hypoxic microenvironment acts as a trigger for autophagy. Autophagy functions to maintain low levels of intracellular ROS. The intricate interplay between autophagy and ROS levels determines the fate of stem cell differentiation into preadipocytes. Conversely, the interplay between autophagy and ROS influences the transcriptional regulation of adipose regulatory factors, ultimately affecting the differentiation of preadipocytes. Recently, a research group established a LEPTIN-deletion pig obesity model (114). LEPTIN deletion in pigs causes type II diabetes and non-alcoholic fatty liver disease. Porcine LEPTIN deficiency inhibits JAK2-STAT3 signalling and enhances oxidative stress. JAK2-STAT3 signalling affected the expressions of lipogenic-related SOCS3, SREBP1c, ACSL3 and ACSL5. Moreover, LEPTIN knockout activates the AMPK signal and enhances mitophagy. Notably, there are no significant changes in the activation of mTOR, MAPK and PI3K-AKT pathways. More surprising is that LEPTIN-deficient rat livers are void of hepatic fibrosis, mitochondrial autophagy and oxidative stress. By comparing these results, they found that the phosphorylation level of AMPK was responsible for these differences. Based on these studies, it can be inferred that Nrf2, p38, mTOR, AMPK and FOXO1 may serve as key nods between autophagy and oxidative stress during adipogenesis. There may exist other pathways linking autophagy and oxidative stress. For instance, β-cypermethrin promoted adipogenesis via oxidative stress-mediated autophagy disturbance, and it caused macrophage polarization by mediating miR-34a (115). Arsenite treatment induces oxidative stress by decreasing UCP1 expression. Meanwhile, arsenite inhibited autophagy necessary for the homeostasis of brown adipose tissue by suppressing Sestrin2 and ULK1 (116). However, the specific signalling regulation has not been further investigated. More research is needed to identify novel targets linking autophagy and oxidative stress in adipogenesis.

### 5.2 The adipogenesis is regulated by autophagy-inflammation crosstalk

**Figure 2:**
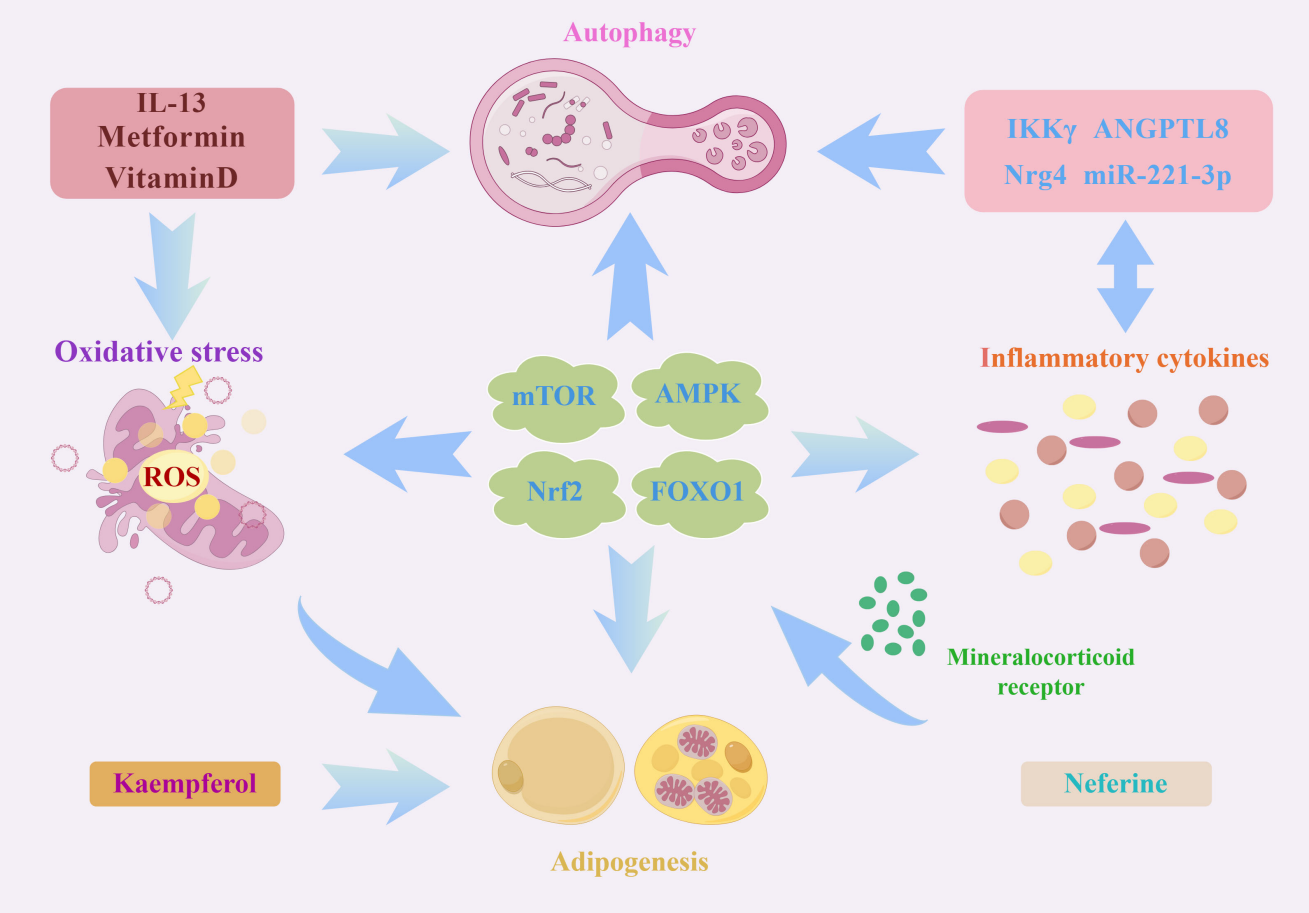
A schematic illustration depicting the intricate interplay between autophagy, oxidative stress, and inflammation in the process of adipogenesis. As an important intracellular energy receptor, AMPK is the most critical node linking autophagy, oxidative stress and inflammation. On the one hand, AMPK can affect the expressions of lipogenic genes (including SOCS3, SREBP1c, ACSL3 and ACSL5) through JAK2-STAT3 signalling. On the other hand, AMPK can regulate different autophagic processes through specific phosphorylation regulation of ATGs, such as ULK1 and Beclin-1. In addition, AMPK affects the expression of various oxidation-related proteins such as Nrf2, SOD and UCP2. Moreover, the activated AMPK is also involved in ameliorating inflammation by mediating NF-κB activation. Similarly, Nrf2 and PI3K/Akt/mTOR signalling pathways are also worthy of attention. Several molecules, such as MR and neferine, affect the autophagy-oxidative stress-inflammation axis through these pathways. In addition, some autophagy proteins, ATG7, TP53INP2 and autophagy negative regulator Rubicon, can regulate adipogenesis by impacting PPARγ activation. All these signals and molecules are important factors in the regulation of autophagy and redox equilibrium and participate in the transcription of many lipogenic genes and pro-inflammatory cytokines.

Autophagy represents a vital process for maintaining homeostasis, exerting diverse effects on the immune system. Notably, autophagy has the potential ability to regulate inflammation, which has systemic implications and directly impacts the development of both innate and adaptive immunity, thereby influencing various disease states and cellular physiological conditions (117). On one hand, the regulation of autophagy is influenced by pro-inflammatory cytokines. While on the other hand, it functions to restrain excessive inflammatory responses. Metformin and vitamin D modulate adipogenesis by impeding the formation of autophagosomes and suppressing adipose inflammation, thereby hindering adipogenesis in WAT and facilitating the differentiation of BAT. Nevertheless, this context’s mutual regulation between autophagy and inflammation remains elucidated (118). Members of the Angiopoietin-like protein (ANGPTL) family serve as natural inhibitors of lipoprotein lipase and play a crucial role in lipoprotein and triglyceride metabolism in response to nutritional cues. ANGPTL8 has been implicated in NF-κB-mediated inflammation, autophagy, adipogenesis, intracellular lipolysis, and circadian rhythm regulation. Inflammations can downregulate ANGPTL8 expression in human adipocytes by enhancing the levels of miR-221–3p. A strong association was observed in a murine model between the cycling of ANGPTL8 and lipopolysaccharide-induced acute inflammatory responses i. Further studies have verified that ANGPTL8 negatively regulates NF-κB activity by facilitating the kinase IKKγ autophagic degradation (119). Another bioflavonoid, Kaempferol, has been demonstrated to activate autophagy in osteoblasts through the inhibition of adipogenesis, reduction of inflammation and oxidative stress, as well as promotion of osteoclast autophagy and survival, thereby achieving osteoprotective effects. The mitigation of oxidative stress and inflammatory responses through autophagy represents a critical step in preventing and treating osteoporosis (120). Additionally, while neuregulin4 knockdown does not appear to impact adipogenesis, studies have revealed its detrimental effect on the insulin responsiveness of 3T3-L1 adipocytes. Moreover, neuregulin4 knockdown has modulated the expression of pro-inflammatory cytokines and autophagic degradation (121). Adipocyte-specific depletion of TBK1 mitigates obesity induced by a high-fat diet by augmenting energy expenditure. Subsequent investigations reveal that TBK1 directly impedes AMPK, suppressing respiration and enhancing energy accumulation (122). Conversely, activating AMPK under catabolic circumstances promotes the phosphorylation of TBK1 through its downstream effector, ULK1. More surprisingly, TBK1 knockout also exaggerates adipose tissue inflammation and insulin resistance. TBK1 exerts its anti-inflammatory effects by phosphorylating and provoking the degradation of the IκB kinase NIK, consequently inhibiting NF-κB activity. In addition, TBK1-mediated AMPK activity hurt NF-κB activation. These studies highlight a significant interplay between inflammation, autophagy, and oxidative stress in adipogenesis. However, there remains a dearth of comprehensive investigations analysing the synergistic mechanisms contributing to adipogenesis. Based on this research, it is reasonable to speculate that mTOR, AMPK, FOXO1, and NRF2 signalling pathways may serve as pivotal nodes that interconnect autophagy, oxidative stress, inflammation, and adipogenesis (**Figure 2**).

## 6 Summary and perspectives

Adipogenesis is a process influenced by multiple factors. Studies have identified autophagy, oxidative stress, and inflammatory factors as key regulators of adipogenesis, which participate in regulating these pivotal signals. Autophagy protein ATG7, TP53INP2, and autophagy negative regulator Rubicon all impact PPARγ activation (63, 65, 123). Furthermore, cellular autophagic flux is a critical determinant of MSC adipogenic differentiation (124). In turn, these adipogenic factors can also regulate adipogenesis by targeting autophagy-related genes (such as Beclin-1, LC3B, and p62) and autophagy transcription factors (including FOXO1, TFEB, and XBP1) (57). Cytokines TNF-α, IL-4, IL-6, IL-7, and IFN-γ inhibit adipogenesis through diverse mechanisms, including activation of the Wnt/β-catenin/TCF-dependent pathway or inhibition of PPARγ and C/EBPs (8, 125). Furthermore, MCP-1 induces autophagy and endoplasmic reticulum stress during the initial phase of adipogenesis. This is accompanied by increased expression of adipocyte differentiation factors such as C/EBPs and PPARγ (126). Sustained ROS stimulation alters mitochondrial dynamics in adipocytes, decreases cellular respiration efficiency, and downregulates the expression levels of PPARγ, C/EBPα, and the lipogenic marker PLIN1 via Nrf2 or SIRT1 signalling, resulting in impaired adipogenesis (12, 13, 127). Inhibition of autophagy, oxidative stress, and inflammation reduces adipose tissue mass and impacts obesity. Moreover, there is evidence that autophagy inhibitors, ROS scavengers, and pro-inflammatory cytokine antagonists contribute to regulating adipogenesis and enhancing lipid metabolism (128–130). Therefore, combined targeting of autophagy, inflammation, and oxidative stress is a promising therapeutic avenue for obesity and its associated complications.

However, autophagy, inflammation and oxidative stress are double-edged swords in many physiological processes. Many lines of evidence show that autophagy, inflammation, and oxidative stress can either promote adipogenesis or induce dyslipogenesis. Although most research suggests that autophagy and oxidative stress predominantly facilitate adipogenesis, a few contrasting findings persist. One study found that when autophagy initiation-related protein ULK1 was knocked down in 3T3-L1. However, autophagy was significantly inhibited, adipogenesis was not affected, and the expression of transcription factors (C/EBPs and PPARγ) in adipose differentiation was not significantly changed. The study suggests that ATG5-dependent autophagy, rather than ULK1-dependent autophagy, may be critical for adipogenesis (26). Similarly, ROS can impede adipogenesis by heightening the inhibitory factor of lipogenesis, CHOP-10/GADD153 (85). Additionally, the influence of diverse inflammatory factors on adipogenesis exhibits considerable variability (8). Adipogenesis is a very complex process involving numerous events, such as cell fate determination, energy metabolism and physiological homeostasis. Based on the characteristics of adipogenesis and the results of many studies, we believe that the autophagy-oxidative stress-inflammation axis plays a key role during adipogenesis. Autophagy, oxidative stress, and inflammatory response are intricately linked, and their interplay may collectively dictate the course and fate of adipogenesis. Previous studies have usually focused on one of these points, ignoring the fact that adipogenesis is actually the result of multifactorial regulation. An integrated study of the modulation of adipogenesis by multiple factors will be the focus of the next study. Therefore, the identification of signalling pathways or molecules that can comprehensively regulate autophagy, inflammation, and oxidative stress will be important for elucidating the mechanisms of adipogenesis and accelerating the exploration of molecular targets for the treatment of obesity-associated metabolic disorders. Here, we point out that mTOR, AMPK, FOXO1, and Nrf2 may be promising targets that simultaneously affect autophagy, oxidative stress and inflammation during adipogenesis. Continued research into the specific mechanisms by which these nods integrally regulate adipogenesis is a significant work. A thorough exploration into the roles of autophagy, inflammation and redox homeostasis in adipose tissue and their interaction carries profound implications for elucidating the mechanisms of adipogenesis and expediting the exploration of molecular targets for treating obesity-related metabolic disorders.

## Author contributions

CHo: Writing – original draft, Writing – review & editing. XL: Writing – original draft, Writing – review & editing. KZ: Writing – original draft, Writing – review & editing. QH: Writing – original draft, Writing – review & editing. BL: Writing – review & editing. HX: Writing – review & editing. BH: Validation, Writing – review & editing. FM: Writing – review & editing. XZ: Writing – review & editing. DT: Writing – review & editing. CHu: Writing – review & editing. CT: Writing – review & editing. LJ: Writing – review & editing. YC: Writing – review & editing. HW: Writing – review & editing. BD: Writing – review & editing. SW: Writing – original draft, Writing – review & editing.
